# Through babies’ eyes: Practical and theoretical considerations of using wearable technology to measure parent–infant behaviour from the mothers’ and infants’ view points

**DOI:** 10.1016/j.infbeh.2017.02.006

**Published:** 2017-05

**Authors:** R. Lee, A. Skinner, M.H. Bornstein, A.N. Radford, A. Campbell, K. Graham, R.M. Pearson

**Affiliations:** a Centre for Academic Mental Health, School of Social and Community Medicine, University of Bristol, United Kingdom; b MRC Integrative Epidemiology Unit at the University of Bristol, United Kingdom; c Child and Family Research, *Eunice Kennedy Shriver* National Institute of Child Health and Human Development, MD, USA; d School of Biological Sciences, University of Bristol, 24 Tyndall Avenue, Bristol BS8 1TQ, United Kingdom

**Keywords:** First person view, Dyadic interaction, Behaviour, Demand characteristics, Wearable, Camera

## Abstract

**Aims:**

To explore the utility of first-person viewpoint cameras at home, for recording mother and infant behaviour, and for reducing problems associated with participant reactivity, which represent a fundamental bias in observational research.

**Methods:**

We compared footage recording the same play interactions from a traditional third-person point of view (3rd PC) and using cameras worn on headbands (first-person cameras [1st PCs]) to record first-person points of view of mother and infant simultaneously. In addition, we left the dyads alone with the 1st PCs for a number of days to record natural mother–child behaviour at home. Fifteen mothers with infants (3–12 months of age) provided a total of 14 h of footage at home alone with the 1st PCs.

**Results:**

Codings of maternal behaviour from footage of the same scenario captured from 1st PCs and 3rd PCs showed high concordance (kappa >0.8). Footage captured by the 1st PCs also showed strong inter-rater reliability (kappa = 0.9). Data from 1st PCs during sessions recorded alone at home captured more ‘negative’ maternal behaviours per min than observations using 1st PCs whilst a researcher was present (mean difference = 0.90 (95% CI 0.5–1.2, *p* < 0.001 representing 1.5 SDs).

**Conclusion:**

1st PCs offer a number of practical advantages and can reliably record maternal and infant behaviour. This approach can also record a higher frequency of less socially desirable maternal behaviours. It is unclear whether this difference is due to lack of need of the presence of researcher or the increased duration of recordings. This finding is potentially important for research questions aiming to capture more ecologically valid behaviours and reduce demand characteristics.

## Highlights

• Ecologically valid measurement of mother–infant behaviour is difficult.
• Observations often rely on short snapshots with a researcher present.
• Wearable cameras can be worn by mothers and infants alone at home.
• Home interactions were compared with an observation with a researcher.
• More negative maternal behaviour was recorded using ‘headcams’ alone.

## 1 Introduction

Variations in mother–infant interactions have a substantial impact on offspring health and functioning in later life. Non-human animal studies have demonstrated stable and enduring changes in the brain as the outcome of variations in maternal behaviour, even in cross-fostering studies which eliminate the influence of genetic transmission (Francis, Diorio, Liu, & Meaney, 1999). A recent human study also demonstrates associations between variation in parenting within the normative ranges and infant brain development (Bernier, Calkins, & Bell, 2016). Experimental manipulations in human mothers further demonstrate the causal role of maternal behaviour on infant and child development. The still-face procedure (Cohn & Tronick, 1983), where the mother is instructed to behave temporarily in a disengaged manner (blank face and non-response) results in immediate infant distress. In addition, manipulation of contingency of maternal verbalisations (either responding to infant vocalisations within an appropriate time frame or not) leads to changes in infant vocalisations (Goldstein, Schwade, & Bornstein, 2009). Longitudinal studies, which have measured maternal behaviour, also highlight associations between variations in maternal behaviour and longer-term emotional, behavioural, and cognitive outcomes in children (Bornstein, Arterberry, & Lamb, 2014). However, many questions regarding the long-term impact of variations in maternal response remain unanswered. The first step is ecologically valid measurement of parental behaviour, and this is the focus of the current paper.

Measurement of maternal behaviour, in large longitudinal studies or randomised control trials investigating parenting interventions, is essential to understanding parental behaviour and its effects. The accepted gold standard for measuring mother–child interactions is generally to have a researcher observe or film an interaction between mother and child in a clinical, research, or home setting and film from this third person point of view (3rd PC). There are several limitations to this approach, however:

1. Demand characteristics or reactivity

Observation from a third, often unknown party (the researcher) is undeniably intrusive (Heisenberg, 1927). In observational study, the presence of a videographer may represent a kind of novelty that evokes atypical responses from those observed; this phenomenon is termed “reactivity” or ‘demand characteristics’. Observation may promote socially desirable or appropriate behaviours and suppress socially undesirable or inappropriate behaviours (e.g., adults may display higher rates of positive interactions with children; Baum, Forehand, & Zegiob, 1979; Zegiob, Arnold, & Forehand, 1975). This result may be differential according to different maternal characteristics; that is, some mothers may behave more positively, whilst others may become self-conscious and thus behave less positively (Weber & Cook, 1972).

2. False representation of the infant's experiences

Generally, if maternal behaviour is coded from the viewpoint of an observer (3rd PC), what is coded is what the observer sees and not necessarily what the infant or mother experiences. From the point of view of developmental research, however, the ideal is to capture the infant's or mother's experience. For example, a mother smiling at her baby while the baby is looking at the floor differs from when the infant actually sees the smile. Whilst in both cases the intent may be the same and the smile is an act of warmth by the mother, from the infant's point of view the maternal behaviour is unlikely to influence the child if the child misses it.

3. Participant and researcher burden

Due to demands on participant and researcher time, observations are usually of short duration and therefore only provide a snapshot of the mother–child relationship.

### 1.1 Current study: first-person viewpoint

First person cameras (1st PC) are small portable cameras worn by the participant facing outward to capture the view point of the individual. A few recent studies have used 1st PCs, worn on head bands in an effort to capture the viewpoint of mothers and infants. Bornstein and Arterberry (2010), for example, used infant and mother worn 1st PC to record and compare the world from infant versus adult perspectives. Yurovsky, Smith, and Yu (2013) also used this technique to assess how infant's co-ordinate attention with a visual partner and highlight the different viewpoints of babies as compared to adults. For example, a child's view largely consists of a single dominating object compared to a mother's wider perspective. Sugden, Mohamed-Ali, and Moulson (2014) also used 1st PCs worn by infants for a number of hours at home, in order to measure the infant's exposure to adult faces

Using 1st PCs offers many practical and research advantages that address the three shortcomings listed above. They: (1) eliminate the need for a researcher to be present, reducing potential influences of the researcher on mother and infant behaviour; (2) record the viewpoints of each interactant, so different perspectives are captured; and (3) diminish participant burden by removing the need to attend or host a research visit. So far, however, these studies have only measured the viewpoint of one-half of the dyad and therefore miss the combined footage. In the present paper, we explore and evaluate the gains and limits of using 1st PCs simultaneously worn both mother and infant. We evaluate how well 1st PCs capture relevant information via video and audio data-collection functions, we describe the reliability of existing coding systems that could be used on data captured by 1st PCs, and we explore 1st PCs ability to attain the 3 advantages described above.

We first investigated whether two independent raters show reliability when coding behaviours from video footage from 1st PCs alone. This would mean that the recorded footage from 1st PCs alone is of adequate quality that it can be reliably coded as the same event by two different coders.

We next explored whether the 1st PC reduced the role of participant reactivity (advantage number 1). We hypothesise that removing the researcher and allowing a longer duration will reduce reactivity and demand characteristics thereby reducing a fundamental bias in observational psychological research. We predict that, for mothers and infants left alone with the 1st PCs *without* the researcher present over a number of days, there will be a more negative in the types of behaviours recorded as compared to interactions recorded by the 1st PC but *with* a researcher present. For this comparison we keep the camera view point constant but vary the presence of a researcher. We specifically hypothesise that we will see a greater frequency of less socially desirable maternal behaviours, such as distracted and critical responses.

We finally explored the potential advantage of recording from the infants’ view point (advantage number 2) by documenting the concordance of behavioural coding of footage from 1st PCs with footage from 3rd PCs, when recording the *same interactio*n and varying only the *camera view point*. High concordance would mean that the majority of the same information is picked up by both viewpoints and is interpreted in the same way by coders. Low concordance could indicate that one of the recording methods picks up unique (and potentially important) information. We explored sources of differential concordance to understand information that is potentially lost or gained from using the different viewpoints.

## 2 Method

### 2.1 Participants

Mothers with infants between 3 and 12 months of age were recruited using email advertisements within the University of Bristol School of Social and Community Medicine social life email list to Staff (academic and admin) and PhD students. Fifteen participants were recruited. Mean maternal age was 32.3 years (*SD* = 4.7), and mean age of infants was 8 months (*SD* = 2.4). All mothers were married or cohabiting and had high levels of education (at least one degree); all but one participant was Caucasian.

### 2.2 Procedures

Infants were placed on a play mat with a selection of the same set of age-appropriate small, soft, and plastic toys, and mothers were asked to play with their infants as they would normally. Mothers and infants were filmed by a researcher, and both wore 1st PCs that recorded play during this time (see materials). The observation lasted 11 min.

The researcher then instructed the mother how to record with and charge the 1st PCs, and asked mothers to use them during a variety of play times, meal times, and bed times in the coming days. The researcher left the 1st PCs with the mothers for an average of 1 week depending on the participant's availability. Participants were asked to record at least three 30-min sessions.

Mothers were given a packet with instructions on how to use the 1st PCs, a session diary to record when they had filmed sessions, and a questionnaire about how they found using the 1st PCs (see Appendix). Finally, mothers were asked to complete a standard demographic questionnaire to provide information on highest educational level, occupation, age, number of children, and current feeding method.

### 2.3 Materials

We captured video and audio footage of mother–infant interactions using low-cost, head-worn cameras that have previously been used for recording infant's eye views of their environment (Sugden et al., 2014). The particular 1st PC we used was a ‘Bogdan Digital Spy Hidden Camera DVR Video Recorder’, at a cost of approximately £20 per camera, plus £5 for an SD storage card for each camera. The 1st PCs record video and audio in AVI format. Video has a resolution of 720 × 480 at 30 frames/s. No specification is provided for audio bitrate or sampling rate. 1st PCs store video on an SD card of up to 16 GB capacity or approximately 1 h of video and audio. The 1st PC has an internal Li-Ion battery, which gives approximately 2 h of battery life.

1st PCs are marketed as novelty spy cameras in the form of lapel badges and are yellow in colour with a black smiley face. To reduce the probability of the infant's gaze being unduly attracted to a brightly coloured 1st PC attached to the mother, we coloured them black. To attach the 1st PCs to the head of the mother and infant, they were sewn into elastic headbands.

**Fig. 1:**
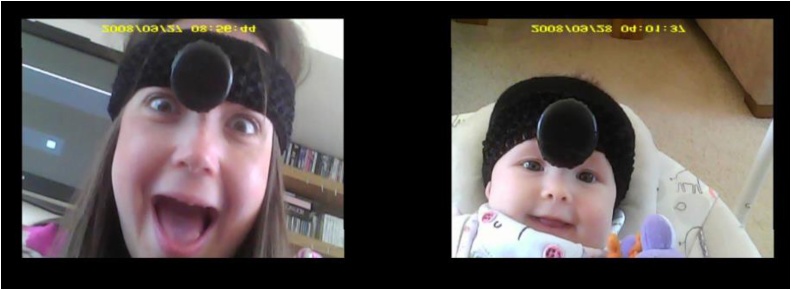
Mother and baby combined footage (with permission).

Footage from the two 1st PCs (mother and infant) was synchronised in time according to an early common event in both cameras and then joined using light works video editing software. The combined footage was then coded as below (see Fig. 1 for an example of combined footage).

### 2.4 Coding

Observations were coded using Noldus Observer XT software to categorise each maternal behaviour (event-based coding), using existing, published operational codes of basic maternal behaviour and infant affect (Leerkes, 2010). Mother codes were mutually exclusive, so at any one time point mothers can only be coded in one category. The codes are also exhaustive, so every maternal event is coded, and the duration of this code was automatically recorded. The coding categories were comforting, engagement, encouragement, positive affect, monitoring, routine care, distracted, critical, mismatched affect, persistent ineffective, and intrusive. Full description is available from Leerkes (2010), and we provide a brief description of each code here:

### 2.5 Positive maternal behaviours

Comforting: involves any maternal behaviour that will make the infant feel more at ease, such as hugging.

Engagement: is when a mother directs her actions towards the infant (e.g. talking to the infant).

Encouragement: consists of any maternal action that is associated with spurring the infant on.

Positive affect: is the display of any positive emotion which is not part of one of the other codes (such as comforting), so for example the mother's comforting her child whilst smiling is coded as comforting, as the primary action relates to the objective to comfort; likewise if the positive emotion is primarily used to encourage, then it is coded as encourage.

### 2.6 Neutral maternal behaviours

Monitoring: this involves the mother watching the infant but not being actively involved.

Routine care: this category encompasses acts such as cleaning the infant or adjusting clothes.

### 2.7 Negative maternal behaviours

Distracted: the mother is neither engaged with nor monitoring the infant, and may be looking away or involved in a separate activity.

Critical: is any maternal behaviour that involves directing negativity towards the infant.

Mismatched affect: is when the mother laughs or smiles when the infant is distressed, wary, and nervous, etc. (does not include attempts to distract or reassure the infant while engaging, supporting or calming). May also include contradicting or denying infant's emotional or behavioural reaction (e.g., “you’re not scared” or “that's not scary” or “it's funny” in matter-of-fact, firm tone if infant is distressed). The coding specifies that if mismatched affect occurs it should be coded rather than any other behaviour with which it potentially co-occurs.

Persistent ineffective: this behaviour involves the mother repeating a behaviour despite the fact that her behaviour does not have a positive influence on the infant's emotional state.

Intrusive behaviour: is when the mother's actions conflict with the infant's desired outcome, so for example preventing the infant from getting a toy reached for.

In the coding manual clear guidance is provided regarding which code to prioritise if two codes co-occur and in which situations to score which code. We used event-based, continuous coding. So, the mother's behaviour would be coded into one of the categories described above The duration of this behaviour would then be recorded in the software until a new behaviour is recorded The video was first coded for maternal behaviours and then separately for infant affect (happy, neutral, or distressed) again with operationalised codes for infant emotion based on Leerkes (2010). Therefore, for each period of time, a code for maternal and infant affect is recorded.

Coders were trained and supervised by RP, two coders were psychology final year placement students (AC and RL) and a final coder was a psychiatrist (KG). A series of training coding sessions to reach reliability of >80% on standard 3PCs was conducted before coding the headcam videos.

### 2.8 Analysis

#### 2.8.1 Inter-method recording concordance on the same observation

Inter-method reliability was calculated using the 1st PC and 3rd PC footage of the same play observation with the researcher present for all 14 dyads (thus 14 pairs of videos). For each the 1st PC and 3rd PC coding were paired, and overall reliability calculated (in The Observer XT 11). As we were interested in the amount of each behaviour as well as the order in which behaviours occurred, the duration/sequence calculation was used with a tolerance window of 2 s (Jansen, Wiertz, Meyer, & Noldus, 2003). For this comparison we used codings from the same coder to ensure that differences between coders did not account for differences between the coding from the different recording methods. As described below, we also ensured that 1st PCs were reliably coded by an independent coder who had not seen the 3rd PC footage.

#### 2.8.2 Inter-rater reliability of coding the 1st PCs

Given the time intensive nature of coding, it is standard practice to only double code only a proportion (10–20%) of videos for reliability (Nicol-Harper, Harvey, & Stein, 2007; Zosuls et al., 2009). A random sample of 20% (11 of 56 1st PCs observations, as each participant gave multiple 1st PC observations) of all 1st PCs were also coded by an independent trained coder who was blind to the hypotheses of the study and who had not seen the 3rd PCs. Reliability between the same 1st PC codings from the two raters was compared in Observer using the same method as described for inter-method reliability.

#### 2.8.3 Comparing frequencies of maternal behaviours within participants but across recording methods

Descriptive statistics were evaluated to provide summaries of coding parameters across video recording method. Due to the small sample and proof-of-concept nature of the study, we were not powered to conduct multiple comparisons. We therefore focus on key hypothesised comparisons and use paired *t*-tests to test specific comparisons between rates of less sensitive behaviours during interactions with and without a researcher present.

## 3 Results

### 3.1 Number of free sessions recorded

Across the 15 mother–infant dyads, 56 free session videos were recorded for total of 14 h of these free sessions. The videos were a mean of 20 min and 12 s long, and each dyad conducted 1–5 sessions. All sessions were recorded during meal times or free play.

### 3.2 Inter-method concordance

Overall concordance was measured for 14 pairs of 1st PC and 3rd PC videos filming the same situation. The range for the index of concordance was 0.84–0.98, with an average of 0.90. Kappa values fell between 0.78 and 0.98, the mean being 0.89.

### 3.3 Inter-rater reliability

Twenty percent (11) of the 1st PC videos were randomly selected and coded by independent researcher (who did not see the 3rd PC videos) to measure inter-rater reliability of the 1st PCs. The index of concordance from these calculations ranged from 0.78 to 0.98, with a mean of 0.91. The more conservative kappa values ranged from 0.75 to 0.97, with a mean of 0.90. A second analysis was conducted in which behaviour modifiers (intensity of the behaviour: as well as showing infant distress, which the coder qualified as mild, moderate, or intense) were included, resulting in a more granular measure of behaviours. Even in this case, the index of concordance ranged from 0.76 to 0.98, with a mean of 0.90, and the kappa values fell between 0.74 and 0.97, with a mean of 0.89.

### 3.4 Descriptive investigation of non-concordant responses between 1st PC and 3rd PC

Whilst the concordance of the footage from different viewpoints was high, it was not perfect and it is important to establish the nature of prominent discrepancies. Due to the time intensity of this in-depth analysis, a randomly selected sample 9 of the 14 pairs (64%) of 1st PC and 3rd PC videos were selected, and discrepancies between their codes (made by same coder originally) were examined by a third independent rater to understand the nature of information lost or gained from 1st PCs. A total of 178 events that were coded differently from the different view-points were found from pairs (as demonstrated from the high concordance reported above, this is a small proportion of all events), and researchers categorised the nature of differences according to whether the behaviour/action related to the code was visible from both viewpoints (3rd PC, and mother and infant 1st PC combined) and to what extent, as well as more descriptive analysis of the likely cause of different coding. Discrepancies consisted of differences in coding behaviours/actions viewed by both cameras (so likely a coding disagreement), actions/behaviours observed only on 1st PCs, and actions/behaviours observed only by 3rd PCs. We discuss the nature of these different discrepancies below.

Approximately one-third (35%) of differences were behaviours/events that were identified on both cameras but coded differently. Thirty-five percent of these discrepancies were due to coding differences despite the action being clear from both viewpoints. However, the majority of miscoded actions potentially resulted from the different points of view offered by the 1st PC and 3rd PC. For example, the behaviours that were most often differently classified were ‘monitoring’ and ‘engagement’, as the level of engagement of mothers was potentially assessed differently when different viewpoints were taken. Monitoring was usually coded from the 3rd PC and not coded from the 1st PC because the whole body of the mother was visible to the 3rd PC and thus her posture and direction of gaze were much clearer from 3rd PC.

Approximately one-half (48%) of the differences were actions that were picked up on the 3rd PCs, but missed on the 1st PCs. These actions were typically whole-body movements. For example, mothers picking up their infants, or infants flapping their arms were generally missed by the 1st PC. On one occasion, for example, a mother was silent on the 1st PC recording and no other changes in behaviour were visible. However, the 3rd PC recording showed that the mother was handing the infant a toy and maintaining eye contact.

Behaviours that were picked up by the 1st PCs, but missed by the 3rd PCs accounted for about one-fifth (16%) of the discrepancies. Generally, the sound quality of 1st PCs was superior to that of the 3rd PCs as 1st PCs were closer to the mouths of the participants. Sounds such as whispering, which could not be heard on the 3rd PC were recorded by the 1st PC. Also, as 1st PCs were focused directly on participants’ faces, facial expressions that were often missed by 3rd PC were visible on 1st PCs. In the majority of cases in this initial work, 1st PCs were not optimally placed by participants, meaning that many facial expressions and other actions may not have been coded. Thus, the 16% is likely to increase with better positioning of cameras. As technology improves and cameras become smaller and less intrusive, more actions that would be missed by a 3rd PC should be easily identified on 1st PCs.

### 3.5 Performance of the cameras

Many aspects of the 1st PCs’ performance were perfectly adequate to the task. The video quality, while not high definition, can discern facial expressions, eye gaze, and general facial and body movements and responses. Likewise, the audio quality is good enough to record speech. Many shortcomings will be rectified with new and developing technologies.

The novelty, low-cost nature of the device means that some aspects of their performance and functionality were sub-optimal. The field of view (which we estimate to be about 60°) for one interactant was often too narrow to fully capture the partner interactant. The usability of the device was poor, with operations (e.g., switching on and off, and starting and stopping recording) controlled by unintuitive combinations of buttons presses.

### 3.6 Overall behaviours from different recording methods

As can be seen in Table 1, the 3rd PC picked up more positive and neutral behaviours per min than the 1st PCs when recording the same interaction. The 3rd PC also picked up more maternal behaviours across the categories than the 1st PCs, when viewing the same interaction. However, the 1st PCs picked up more infant distress perhaps because of the superior audio and close facial recording of 1st PC as described above. Given that infant distress is most commonly expressed in crying vocalisations and clear facial expressions, 1st PCs may have greater potential to pick up these subtle displays.

During the free sessions, 1st PCs picked up significantly more insensitive behaviours, such as intrusive or distracted maternal behaviour, per min than the 1st PCs while the researcher was present (paired *t*-test: *t*_14_ = 5.6, *p* < 0.001; mean difference = 0.90, 95% CI = 0.5–1.2). This difference represents an approximately 1.5 *SD* increase in such behaviours between the sessions with a researcher present and during the free sessions.

## 4 Discussion

### 4.1 Summary of key results

1st PCs recorded information that resulted in similar overall coding on a relatively simple mother–infant coding system to that recorded from 3rd PC. As predicted, once the researcher was not present, the videos contained less sensitive maternal behaviour. Although only a small pilot sample, even in this highly educated low-risk group the differences were of a potentially large size (approximately 1.5 *SD* higher rates per min of less sensitive behaviour). We do not know if this difference was due to the lack of researcher or different scenarios chosen by the mother when using the 1st PC, but either way, following the protocol here we observe a more negative maternal behaviours.

We see fewer behaviours from the 1st PC compared to the 3rd PC, as indicated from the rate per min of maternal behaviours (see Table 1). Two possible explanations present themselves: The restricted camera angle means that 1st PCs miss maternal behaviours that are actually experienced by the infant or 3rd PCs record maternal behaviours not actually seen by the infant. Our descriptive analyses of the different codings suggest that the former may be more accurate. However, some behaviours (such as infant distress) were often picked up only by 1st PCs (see later).

### 4.2 Advantages of 1st PC

The first advantage relates to reduction in participant reactivity and demand characteristics. Even in this relatively homogenous sample of educated mothers, a substantial difference emerged in the amount of insensitive maternal behaviours over a number of interactions using 1st PCs at home alone than in a one-off researcher present setting, ether with 1st or 3rd PCs. Based on the assumption that mothers are more likely to display more behaviours that are socially considered ‘negative’ parenting when demand characteristics are reduced, the finding of increased negative parenting, provides preliminary evidence for reduced demand characteristics. Demand characteristics may be especially high in educated mothers due to knowledge of research and ‘best parenting’. This potential bias poses a real challenge to measurement of parents’ behaviour, in which consistent associations are made between maternal education levels and observed parenting (see Bornstein, 2015). Our results are consistent with a study demonstrating that a more extreme ‘negative’ maternal behaviour of corporal punishment is recorded more frequently by using passive audio recording in the home than reported frequency from mothers (Holden, Williamson, & Holland, 2014).

There are, therefore, a number of ways in which 1st PCs could lead to reduced demand characteristics. It may because no researcher is physically present, or could result from longer duration of recording. In addition, while the mother is of course still aware that she is being recorded it may be easier to ignore or forget about a camera than the physical presence of a researcher. In addition infants are highly unlikely to be aware that their behaviours will be viewed by someone else while wearing the head cams, and this differs from situations when a researcher is present because infants may be aware of the presence of someone other than their mother, and thus ‘react’. In addition the 1st PC allows a longer duration for the mothers to relax and forget that they are being recorded. Briefer observations can be unstable, and observations lasting longer than an hour are likely to include samples of behaviours from highly varied activities or contexts, thus being more varied themselves (Miller, Shim, & Holden, 1998). It is important to point out that the advantage of reduced demand characteristics is not specific to 1st PC but the home alone setting and other methods (such as stationary cameras in houses) may also offer this advantage and result in more negative behaviours being captured.

Secondly, 1st PCS were better able to capture subtle facial expressions and vocalisations. Assuming 1st PCs are placed at the optimal position (i.e., at the glabella between the participants’ eyebrows), there was also evidence of advantage 2 (view point of the infant). First, the ranges of behaviours that can be captured using a 1st PC differ to those visible on a 3rd PC. Subtle facial expressions and facial expressions that would otherwise be missed due to the 3rd PC camera angles can be recorded on 1st PCs (e.g., Baby FACS; Oster, 2005). Subtle sounds can also be recorded more intelligibly by 1st PCs because their cameras’ microphones are much closer to the face than are 3rd PCs microphones. The viewpoints offered by 1st PCs are unique and possibly more naturalistic, as the visual angle is that of the first person.

Thirdly, the cameras used here have the advantage of reduced costs associated with researcher's time. 1st PCs are less demanding on research time than 3rd PCs as they can be left with participants to record themselves rather than hosting a researcher and associated costs of researcher time and travel.

### 4.3 Challenges

Although there are a number of advantages associated with the use of 1st PCs, there are also some challenges. One relates to placement. In practice, many participants did not place their 1st PCs completely optimally, meaning that many advantages of 1st PC were not fully achieved. Over the course of our pilot, we found that instructing mothers to check the position of 1st PCs in the mirror was key to optimally positioning the head camera. However, even when the camera is placed correctly, the visual field offered by current 1st PCs is restricted, and many whole-body movements are consequently missed. Also, to record facial expressions, two participants must wear the cameras and face one another.

### 4.4 Study types which may benefit from 1st PCS

#### 4.4.1 Evaluation of interventions

Reducing demand characteristics and reactivity may be particularly important in evaluating parenting interventions. Many evaluations of parenting interventions involve filming mothers during free-play sessions, in the presence of a researcher, using a 3rd PC. However, these interventions explicitly focus on teaching parents to implement certain behaviours in certain situations. There is a possibility that mothers ‘perform’ these learnt behaviours when being filmed by members of the same study team who delivered the intervention (even if they are different researchers they may be seen as connected), and this circumstance is highly likely to introduce bias. Using the 1st PCs, researchers can help reduce this bias through removing the researchers’ presence and increasing duration of recordings which is key in accurate evaluation.

#### 4.4.2 Large cohort studies or resource limited studies

Given the low cost and reduced researcher time needed, 1st Pcs may be useful for the large-scale recording of mother–infant behaviour in large epidemiological cohort studies which collect multiple measures and aim to reduce participant burden and save costs. Indeed, the 1st PC are currently being piloted in a large UK cohort ALSPAC, see https://proposals.epi.bristol.ac.uk/?q=node/113441.

#### 4.4.3 Studies focused on facial expressions

Studies interested in facial expressions specifically may benefit from the use of head cameras which capture them well.

#### 4.4.4 Studies interested in infant view point

Researchers interested in understanding aspects of the environment that infants and mothers focus on. Many of the abilities that are thought of as automatic in adults are learned early in life, meaning that infants are challenged by processes that are simple for more experienced individuals and therefore they will focus on different aspects of their environment. For example, the ability to conceptualise and categorise objects improves with experience (Oakes & Madole, 2003), which infants initially find difficult. This means that infants are more likely to focus on objects in their environment (Bornstein & Arterberry, 2010). Therefore, infants’ viewpoints, and consequently information processing, could differ greatly from that of their adult counterparts. With 1st PCs, researchers will have access to more accurate representations of both infant and adult viewpoints, leading to more informed inferences about infant experiences.

#### 4.4.5 Use in video-feedback interventions

In the specific mother–infant context, another possible application of 1st PCs is facilitating video feedback interventions, which have previously been shown to be effective in improving maternal sensitivity in a range of populations, including adolescent mothers, mothers with schizophrenia, and mothers with poor attachment styles (Bakermans-Kranenburg, van, & Juffer, 2003; Cassibba et al., 2015 Cassibba, Castoro, Costantino, Sette, & Van Ijzendoorn, 2015; Kalinauskiene et al., 2009, Reddy et al., 2014). This therapeutic approach consists of video feedback intervention sessions in which mothers are recorded interacting with their infants in a naturalistic free-play session. These recordings are then discussed with a mental health professional, and mothers are encouraged to observe their own sensitive and insensitive behaviours, thereby improving their observational skills and empathy. Any sensitive behaviours displayed by the mother are supported and are used as examples to contrast with instances in which insensitive behaviours are displayed. This means that each mother acts as her own model. A key focus of video feedback is ‘mind-mindedness’, referring to a mother's ability to see the world from her baby's view point and thus respond to the baby's emotional needs (Meins, 1997). 1st PCs would be particularly useful in facilitating mind-mindedness as the infant's actual viewpoint would be captured, and mothers may be better able to understand their infant's world and their infant's perceptions of that world. In addition the mother can reflect on her own responses more easily when viewing the situation from her own view point. This process – known as “self-entheaty” or observing one's actions on record – may be particularly beneficial in a therapeutic sense because psychological and behavioural-change programmes rely heavily on introspection. As Lahlou (2011) brings to light, introspection is not easy, and when we do introspect we change the very cognitions we are hoping to describe. Subjective Evidence-Based Ethnography (SEBE; Lahlou, 2011) allows patients to view their 1st PC data after collection and engaging with the task, in an attempt to examine cognitions after the act. In this way, individuals can avoid the problem of modifying cognition with introspection and gain increased insight by experiencing something and learning independently, as opposed to didactically.

### 4.5 Study types which may benefit from 3rd PCs

#### 4.5.1 Studies interested in global environment and whole body

If more precise and whole-body movements are of key importance, 1st PCs may not be recommended because these behaviours were often missed by 1st PC. Rather cameras in rooms (but without researchers present) could be used alongside the 1st PC to capture whole body movements such as touching or assessing the positioning and proximity of the mother and child. Furthermore, 1st PCs can provide important information regarding the location of dyads within a room. In addition studies interested in multiple participants, such as those including fathers and siblings, may need to include the wider footage captured from 3rd PCs placed in homes.

### 4.6 Future directions

There is considerable interest in on-body camera technology from a variety of domains. Police officers in a number of regions of the United Kingdom and United States are routinely being issued body cameras for recording their interactions with the public. Social care workers in some areas use on-body cameras for similar purposes, providing visual and audio records of meetings with service users. The market sector driving the biggest changes in on-body camera technology is the domestic desire for wearable action cameras. Manufacturers such as GoPro are bringing devices to market that are smaller, lighter, have wider fields of view, higher definition video, longer battery life, and increased flexibility in streaming and storing captured footage to other devices and networks. These improvements mean that a number of devices coming to market will be better suited to on-body video and audio recording of interactions in dyads for psychological research. Future studies of the kind reported here will undoubtedly benefit from these technological improvements, in particular, wider field of view, greater ease of use, longer battery life, greater storage capacity, and ease of video/audio data transfer.

## 5 Conclusion

The high level of concordance between 1st PC and 3rd PC videos of the same situation demonstrates that 1st PCs capture a situation reliably. Some elements of these situations, such as whole-body movements, are missed by 1st PCs because of the relatively small field of vision of the 1st PC. Despite the fact that the audio and visual quality of 1st PCs are at this point still sub-optimal, they capture subtle sounds and facial expressions that may be missed by 3rd PCs. As new technology emerges and cameras with better visual and audio quality and wider fields of vision come onto the market, the performance of 1st PCs should improve. Another potentially important (if preliminary) finding, associated uniquely with 1st PCs, is the increase in insensitive behaviours seen in mothers when they were left alone with 1st PCs. This result suggests that researchers are highly likely to capture an unrepresentative picture of maternal behaviour if researchers are present, even in the home setting. The other main advantages of using 1st PCs are their ease of use, low cost, and the relatively small participant and researcher burden. Future directions may include the use of 1st PCs in video feedback interventions and in developmental and psychological research.

## Figures and Tables

**Table 1:** Comparisons of rates per minute of mother and infant behaviours according to recording method and scenario.

Mean rate per min (SD)	Infant			Mother		
	Happy	Neutral	Distressed	Sensitive behaviours^a^	Neutral^b^	Less sensitive behaviours^c^
Initial test session (3rd PC)	0.72 (0.55)	0.93 (0.59)	0.43 (0.52)	2.13 (1.5)	1.12 (0.9)	0.21 (0.33)
Initial test session (1st PC)	0.52 (0.34)	0.82 (0.29)	0.63 (0.65)	1.35 (0.88)	0.73 (0.44)	0.13 (0.16)
Free sessions (1st PC)	0.50 (0.51)	0.70 (0.45)	0.48 (0.40)	1.24 (0.55)	0.78 (0.78)	1.04 (0.63)

a Comforting, engagement, encouragement, positive affect.

b Monitoring, routine care.

c Distracted, critical, mismatched affect, persitent ineffective, intrustive.
