# Supplementary material for: Through babies’ eyes: Practical and theoretical considerations of using wearable technology to measure parent–infant behaviour from the mothers’ and infants’ view points
Source: Infant Behav Dev. 2017 May;47:62–71. doi: 10.1016/j.infbeh.2017.02.006 (PMC5429397; doi:10.1016/j.infbeh.2017.02.006)

Head camera instructions (online only)

In order to record a session press down on the button on top of the camera case for 3 seconds and ensure that the initial blue light on the right side of the button, (when viewing from the back) changes to an orange light.

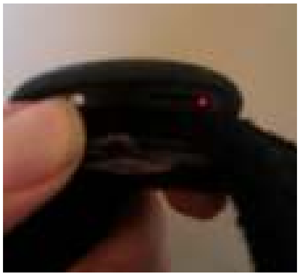

Next, hold down the button to the left for 3 seconds, until the light turns off. The camera is now recording.

Now position the headbands around the top of the head with the buttons face ***down*** in the centre of the forehead, pulled down so that the band is covering the eyebrows but not obscuring vision.

****Please check the positioning of your own camera in a mirror to ensure that the camera is in the lowest position that is comfortable, and in the centre of your forehead. *****

We ask that you try to keep cameras in the same position throughout recording sessions.

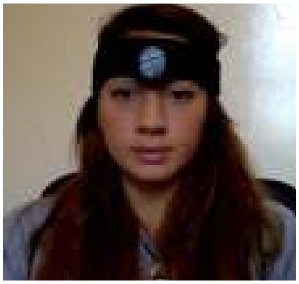

To end a recording please remove the headbands and again press the power button for 3 seconds. The light should flash orange and then disappear.

If your child becomes irritated by the device or falls asleep please remove the headband. Also remove the headband if you think it is necessary for any other reason.

DO NOT LEAVE YOUR CHILD UNNATENDED WITH THE DEVICES

Please charge the devices after each session. In order to charge the devices simply attach the charger to the bottom of the camera case and the other end into a computers USB socket and leave until the next session.

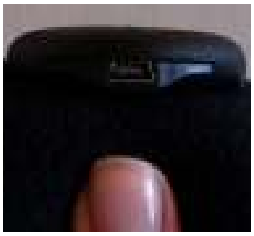

Supplement: Supplementary file 1 [file mmc1.pdf]
